# Nutrition Therapy in Managing Pregnant Women With Gestational Diabetes Mellitus: A Literature Review 

**Published:** 2018-06

**Authors:** Neda Dolatkhah, Majid Hajifaraji, Seyed Kazem Shakouri

**Affiliations:** 1 Physical Medicine and Rehabilitation Research Center, Aging Research Institue, Tabriz University of Medical Sciences, Tabriz, Iran; 2 National Nutrition and Food Technology Research Institute, Shahid Beheshti University of Medical Sciences, Tehran, Iran

**Keywords:** Pregnancy, Gestational Diabetes Mellitus, Medical Nutrition Therapy

## Abstract

**Objective:** Gestational diabetes mellitus is the most common metabolic and endocrine perinatal complication and is a growing health problem worldwide. Considering the fetal programming and its contribution as one of the evolutionary origins of human diseases, it is very important to improve the glucose metabolism in pregnant women, determination of other nutrients, preventing excessive accumulation of fetal fats, emphasis on weight loss measures before pregnancy, dietary intake with low-fat healthy food and prevention of abundant weight loss. In this paper, we have provided a brief review on dietary intake and dietary interventions in GDM from the perspective of nutrition science attending the physiopathology and etiology of the disease.

**Materials and methods:** Electronic search for English and Persian articles has been perform in databases, including Google Scholar, PubMed ,Scopus, Cochrane central ,Science direct, ISC, SID, Magiran, Iran Medex, and Med Libby key words: gestational diabetes, gestational diabetes mellitus, nutrition, macronutrient, micronutrient, Diabetes. All available articles (cross-sectional, descriptive-analytic, and clinical studies with desirable design and review quality studies were used. Reference books including Krause's Food and the Nutrition Care, The Williams Obstetrics editions of the 14^th^ (2017) and the 24^th^ edition (2014) were also reviewed.

**Results:** Nutrition therapy and physical activity are the initial treatment of GDM. Proper and flexible methods of nutrition therapy that successfully regulate maternal glycaemia while improving expected fetal growth have extensive concepts. Meanwhile, dietary supplements with proven beneficial effects can play an important role in improving deficiencies and improving the metabolic profile of patients.

**Conclusion:** Nutritional management is the main treatment for gestational diabetes mellitus and overweight/obesity is the principal contest in patient counseling and interventions during pregnancy. Despite extensive researches carried out, this field is an active research area and requires more clinical research to minimize maternal and fetal complications.

## Introduction

Pregnancy is a complex metabolic condition that includes significant changes in the humoral environment, as well as changes in adipokines and inflammatory cytokines. Pregnancy is associated with a significant increase in levels of estrogen, progesterone, prolactin, cortisol, growth hormone, and oxidative stress indices of TNF-α, fetal, and leptin. Reducing adiponectin from the second trimester intensifies mother insulin resistance to facilitate fetal feeding (1). Gestational diabetes mellitus is defined as glucose intolerance, which is first recognized during pregnancy (2). The condition is a metabolic and endocrine disorder and occurs when the pancreatic function in the pregnant mother is not sufficient to overcome the diabetic condition of pregnancy (3), it is considered as pre-diabetic, and by playing a key role in increasing the incidence of fasting diabetes mellitus, it is one of the predictors of type 2 diabetes in future (in mothers and children from these pregnancies) (4).

In the next 30 years, a significant increase in the number of diabetic patients worldwide to 366 million is expected, and preventive measures must be planned and implemented to prevent this global problem (5). Pregnancy is likely to be a critical period for appropriate interventions and actions aimed at reducing the incidence of type 2 diabetes (6). The prevalence of gestational diabetes mellitus is increasing rapidly in many developed and developing countries (7). The prevalence of gestational diabetes mellitus varies from 1-14% during pregnancy, which depends on the region and nature of the population, different methods of data collection, the non-accidental choice of mothers and the diagnostic criteria used (8). Based on the results of systematic review and meta-analysis by 9% / Mirie et al. in Iran, a total of 9.4% (Confidence interval 95: 8.5-9.3%) of pregnant women are affected (9). In the latest study by Manafi and colleagues, the prevalence of gestational diabetes mellitus is reported to be 11.9% in the northwest of the country, suggesting an increasing trend in prevalence in Iran, probably with increasing age and maternal body mass index (10). In complicated pregnancies with diabetic mother, there is a risk of multiple complications in fetus and mother, which can be prevented by controlling the level of maternal blood sugar during pregnancy and even childbirth (11). The purpose of this review article is to review the recent studies from a nutritional point of view on the control and management of gestational diabetes, according to the physiopathology of this disease with the aim of preventing the short-term and long-term complications.

## Materials and methods

The search for published articles in this field was carried out by researchers through reviewing Persian articles in the Jahad Daneshgahi (ww.magiran.com, www.sid.ir, www.iranmedex.com and reviewing English articles by referring to Science Direct, PubMed, and Scopus by key words: gestational diabetes, gestational diabetes mellitus, nutrition, macronutrient, micronutrient, Diabetes. All available articles (cross-sectional, descriptive-analytic, and clinical studies with desirable design and review quality studies related to gestational diabetes mellitus and the role of macronutrients and micronutrients) were also used. Reference books including Krause's Food and the Nutrition Care, The Williams Obstetrics editions of the 14th (2017) and the 24th edition (2014) were reviewed. 

***Eligibility criteria:*** 1- Articles that have their full text available. 

2- Articles and books published between 1995 and 2018 (1374-1397).

3- Studies published in English or Persian.

## Results

***Pathophysiology of gestational diabetes mellitus:*** Gestational diabetes mellitus is caused by a disorder of at least three aspects of metabolism: insulin resistance, insulin secretion and increased glucose production (12). Although the level of insulin secretion in women with gestational diabetes, like women with normal glucose tolerance, increases, but it is not enough to overcome insulin resistance and maintenance of normal blood glucose levels. This competition, coupled with the reduction of beta cellular deposits, sparks diabetes mellitus (13, 14); therefore, pregnancy is a stress test (13) to induce glucose intolerance and indeed to reveal a genetic predisposition to type 2 diabetes due to humoral changes (15). This is often the case in the second half of pregnancy, so that insulin resistance progressively increases until delivery (7, 16).

***Risk factors for gestational diabetes:*** Gestational diabetes mellitus and type 2 diabetes have similar risk factors and genetic predisposition to a given population. Considering etiology, it is unknown which one is preceded by another (17). Certain factors include: a family history of diabetes, age over 25, obesity, specific ethnic groups (African-American, indigenous Latin Americans, Indians) and previous births of 4 kg or more (macrosomia), and the risk of developing gestational diabetes mellitus in women (17, 18). Women with gestational diabetes have a high risk of developing diabetes in their later pregnancies (19). Some studies have estimated that in 30-70% of the cases, the disease occur in subsequent pregnancies (20, 21).

***Clinical manifestations of gestational diabetes:*** Classical diabetes findings such as high drinking and high urine associated with gestational diabetes are common and often cannot be diagnosed without screening tests (22).

***Diagnosis and screening criteria for gestational diabetes:*** Gestational diabetes has long been clinically diagnosed and there is no standard diagnostic criteria until 1964. Initial diagnostic criteria for gestational diabetes mellitus have been proven by O'Sullivan (23) 40 years ago, and with partial changes has already been used. These criteria identify women at risk of developing diabetes during pregnancy (24). The World Health Organization and Working Group on Pregnancy Studies or IADPSG in 2013 (the International Diabetes Association), a one-step test (two-hour GTT with 75 grams of glucose) was officially approved for all non-diabetic pregnant women (normal and pre-diabetic) for screening and diagnosis of gestational diabetes during the 24-28 weeks of pregnancy. For all pregnant women, a fasting blood sugar test should be requested in the first visit of pregnancy (Table 1).

**Table 1:** Interpretation of blood glucose test results in the first visit of pregnancy    (25)

**Fasting blood sugar (mg/dl)**	≥ normal 93-125 pre-diabetic ≤126 abnormal

According to ADA, diagnosis of gestational diabetes in women could be taken with each of the following criteria (26): fasting plasma sugar of 92 mg/dl and more and less than 126 mg/dl in at any age of pregnancy; 2-hour oral glucose tolerance test with 75 grams of glucose (OGTT) at 24-28 weeks of pregnancy. Definitive diagnosis of gestational diabetes and follow-up is necessary if at least one of the blood glucose test results is abnormal (Table 2).

***Complications of gestational diabetes:*** Gestational diabetes has many harmful effects on mother and fetus. The most common ones are macrosomia, childbirth injuries, cesarean, poly-hydramnious, preeclampsia, neonatal metabolic disorders, and late complications, including type 2 diabetes mellitus of mother in the post-partum period (28).

Hyperglycemia, hyperplasia and hypertrophy stimulate the fetal beta cells, leading to increased insulin secretion and high levels of insulin in the blood. High insulin and glucose result in increased rate of placental metabolism and stimulation of peripheral hematopoietic embryos after birth, accumulation, and neonatal polycythemia (29, 30). Excess insulin can lead to loss of blood glucose and irreversible damage to the brain cells (30). As a result of abnormal glucose metabolism, blood and urine glucose concentrations increase and result in increasing the susceptibility to urinary tract infections (31). In addition to the unpleasant outcomes of diabetes during pregnancy, the history of the disease within 5 years after delivery increases the risk of type 2 diabetes by 18-50% (19, 32). Studies have also shown that gestational diabetes increases the risk of hypertension and dyslipidemia and, as a result, the risk of arterial hypertension and cardiovascular disease in the long term (33-35). Also, studies on the long-term effects of maternal metabolic disturbances on the fetus have shown that the children of mothers with gestational diabetes are prone to impaired glucose tolerance (IGT) and obesity (36, 37).

**Table 2:** Instructions of glucose test results for diagnosis of gestational diabetes         (25-27)

**One-step screening:** **2-hour oral glucose tolerance test with 75 grams of glucose**	
**≥ 92**	Fasting blood sugar (mg/dl)
**≥ 180**	Blood sugar one hour after glucose consumption
**≥ 153**	Blood sugar two hour after glucose consumption
**Two-step screening:** **1- hour glucose tolerance test with 50 grams of glucose (not-fasting)** **3- hour oral glucose tolerance test with 100 grams of glucose**	
**≥ 180**	Blood sugar one hour after glucose consumption
**≥ 95/4**	Fasting blood sugar (mg/dl)
**≥ 180**	Blood sugar 1 hour after glucose consumption
**≥ 154/8**	Blood sugar 2 hour after glucose consumption
**≥ 140**	Blood sugar 3 hour after glucose consumption

***Gestational diabetes and inflammation:*** Proven association between subclinical inflammation and gestational diabetes can be explained through various mechanisms. Progressive insulin resistance occurs as a result of the anti-insulin-like effects of increasing adipose tissue and placental hormones (cortisol and lactogenic human placenta) in gestational diabetes (38). Ultimate glycosylated products, result in increased levels of glucose, increase oxidative stress. They also activate macrophages and increase serum levels of TNF and IL-6, IL-1, resulting from the production of CRP (39). Probably the pro-inflammatory cytokines have a central role in insulin resistance (40, 41). It seems that inflammatory mediators can destroy pancreatic beta cells and their function and, as a result, cause insulin resistance (42, 43). Regarding the central role of inflammation in the pathogenesis of complications of insulin resistance and diabetes, reducing inflammatory cytokines can be effective in preventing these complications (44).

***Gestational diabetes and oxidative stress:*** Pregnancy is a condition of oxidative stress as a result of high metabolic activity in the fetus-placenta. Oxidants have many physiological effects in normal pregnancies, including the advancement and control of cellular fate and plays an important role in natural development through cellular signaling. In the absence of parallel increase in antioxidative activity, oxidative stress is induced. Increased levels of free radicals in gestational diabetes still go up. Evidences of the important role of oxidative stress in the pathogenesis of gestational diabetes (45) and the complications of diabetic pregnancy on mothers and fetuses were achieved (46, 47). The level of oxidative stress can change the duration and severity of the disease side effects. Excessive production of free radicals can lead to extensive cell damage by affecting proteins, DNA and lipids. In systemic oxidative stress, such as mother diabetes, there is a potential for biochemical abnormalities in fetus (48-50). Free oxygen radicals cause inflammation, disturbances in the regulation of metaloproteins and apoptosis. It is likely that oxidative stress management, along with rigid blood glucose control, is useful both before and during pregnancy in women at risk for gestational diabetes, which is a major challenge for researchers and clinicians.

In animal and human studies, most of the researchers reported the relation between gestational diabetes and macrosomia (the most important complication of gestational diabetes) with increased oxidative stress due to decreased anti-oxidant molecules, decreased activities of anti-oxidant enzymes (super-oxide dismutase), glutathione-peroxidase and glutathione-reductase (50-54).

Biri and colleagues reported the dysfunction of antioxidant system and increased activity of malon-dialdehyde (MDA) in cord blood and placental tissue of GDM patients (55). Chen and Schol showed that in these patients, MDA increased, antioxidant enzymes activity decreased and glucose level was positively correlated with MDA concentration (56).

***Gestational diabetes and obesity:*** Obesity plays a major role in the pathogenesis of many medical problems, including metabolic and cardiovascular disease (57, 58). Most of the researchers found that obesity is a kind of mild chronic inflammation (59-62). Inflammatory cytokines, such as CRP, are associated with obesity and consequently, increased risk of insulin resistance, diabetes mellitus, hypertension and dyslipidemia (63-68).

Pre-pregnancy BMI has a significant effect on gestational diabetes. Compared with women with normal BMI, the odds ratio of a woman weighing less than normal was 0.69-0.82): confidence interval of 0.75(95%). The odds ratio of women with overweight, moderate obesity and severe obesity for gestational diabetes are: 1.77-2.19: confidence interval of 1.97 (95%), 2.34-3.87: confidence interval of 3.01 (95%) and 4.27-7.21: confidence interval of 5.55 (95%)(69).

According to Endo et. al findings, insulin sensitivity in obese women with lower glucose intolerance is lower than women with normal weight and insulin sensitivity in pregnant women with gestational diabetes decreases with increasing gestational age (70). Another parameter that is as important as pre-pregnancy BMI is acceptable weight gain during pregnancy. In overweight women during the first trimester of pregnancy, the risk of developing gestational diabetes increases (71, 72).

Greater early pregnancy weight gain had been shown to be associated with increased risk of GDM (73). Antecedent papers have suggested that excessive gestational weight gain (GWG) is associated with harmful maternal and neonatal consequences (74, 75), maternal postpartum fat mass preservation (76) and obesity of offspring (77, 78). In a retrospective study on women with gestational diabetes, in order to examine the relationship between weight gain patterns and blood glucose levels in patients bearing diet, Brustman and colleagues concluded that patients undergoing diet with controlled gestational diabetes and blood sugar levels, showed less weight gain after diagnosis of gestational diabetes than the patients treated with insulin or glyburide. This means that weight gain after diagnosis of gestational diabetes decreases with proper control of blood sugar (79).

It has been shown that nutritional interventions during pre-natal period are effective in improving maternal weight gain (80, 81).

Herring et.al reported that higher levels of weight gain during pregnancy are associated with higher degrees of insulin intolerance in the third trimester of pregnancy (confidence interval of 2.14, 95%: odds ratio (OR) 1.04-4.42: (CI)) (82). According to studies, only 37% of pregnant women had right weight gain and about 30% of them had weight gain higher than the recommendation (83).

Drehmer and colleagues found that overweight in the third trimester of pregnancy is independent of pre-pregnancy BMI and maternal characteristics, with preterm labor and the need for cesarean (84).

About 20 years ago, the Journal of Nutrition for Pregnancy at the Institute of Medicine presented the first weight gain recommendations based on the pre-natal BMI (85).

In women with normal body mass index, total weight gain during pregnancy ranges from 11.4-15.9 kg. This range is 8.6-11.4 kilograms in overweight women. However, obese pregnant women can only weigh up to 7 kg. Recently, epidemic obesity in the United States has been linked to overweight (86), and hence has much attention from health researchers. In 2009, Institute of Medicine (IOM) has published weight gain recommendations for pregnancy. These guidelines are based on the pre-pregnancy body mass index. In women with normal body mass index, the goal is to weigh (11-15 kg) Ib 25-35 and in women with a lower BMI (12-18 kg) Ib is 28-40 weight gain during pregnancy. In overweight patients, target weight gain is (6.8-11.4 kg) Ib 15-25 and in obese women (5-9 kg) Ib 11-20 (87).

 Using the 24-hour dietary recall method, Chang and colleagues concluded that the average daily calcium intake in gestational diabetes mellitus samples is in the range of approximately 1850 and 2300 kcal, which is higher than the mean calorie intake by subjects with normal blood glucose (1596 Kilocalories) (88). In a case-control study to investigate the relationship between dietary habits and nutrient intakes, the average daily calcium intake in mothers with gestational diabetes mellitus was 1959 calories (89). Based on the results of several studies, more calorie intake in pregnant women can increase the risk of diabetes, and in women with gestational diabetes independently of their obesity, may lead to increased insulin resistance and decreased pancreatic beta cell function (90).

***Dietary and macronutrient pattern and gestational diabetes mellitus:*** Observational studies propose that healthy diets before and during pregnancy candecrease the risk of GDM  (91, 92). Researches in the past decade have revealed that improper mother's diet during pregnancy, such as high fat intake, low intake of carbohydrates and fiber, and diet with high glycemic load, increases the risk of developing gestational diabetes (93, 94).

In a cohort study on 3060 Chinese pregnant women whose food intake was evaluated during 24-28 weeks of pregnancy, He and colleagues showed that receiving dietary fiber has a reverse relationship with the risk of developing gestational diabetes (95). On the other hand, a pilot study on women with gestational diabetes mellitus showed that high fiber diets were not associated with lower levels of glucose (96).

There is growing evidence of the positive effects of dietary patterns with high intake of vegetarian foods (such as whole grains, fruits, vegetables, and brains) and fish, and the low intake of processed animal and fatty foods in the prevention and treatment of gestational diabetes mellitus. The mentioned dietary pattern is the Mediterranean diet (Med Diet) (97). It has been shown that a Mediterranean diet is associated with a lower incidence of gestational diabetes and improved glucose tolerance in diabetic pregnant women (98).

According to recent guidelines, pregnant women with GDM should be referred to a nutritionist for medical nutrition therapy (MNT). Specialized MNT is substantial in helping pregnant women with GDM attain and preserve normal serum glycemic levels and proper weight gain while providing essential macro and micro nutrients(99).

***Intestinal microbial environment and gestational diabetes:*** The total genome of the intestinal microbial population encodes 3/3 million unrelated genes that are 150 times larger than the entire human genome. This genetic enrichment enables the intestinal microbiota to possess many active metabolic functions that cannot be addressed by the human genome (100). In recent years, it has been shown that optimum balance in the number of gastrointestinal microbes depends on nutrition and health. The main microorganisms affecting the preservation of this balance are lactobacilli and bifidobacteria (101). Factors affecting the intestinal microorganisms (such as stress and diet) will have an adverse effect on human health by breaking the optimal microbial balance. Medical studies in the past decade have been associated with intestinal microbial population with metabolic disorders, especially diabetes and obesity. The microbial environment of the gut plays a role in planning and controlling of many physiological actions, including the development of epithelium, blood circulation and intrinsic and adaptive mechanisms of the intestine, although not fully understood (102, 103). Pregnancy affects the composition of the intestinal microbial population (104). Generally, at the end of pregnancy, the number of proteobacteria and acinetobacteria increases and bacterial enrichment is reduced (104). These changes have the ability to modify the immune system to facilitate metabolic and immunological adaptation (104, 105). These changes are more pronounced in obese pregnant, overweight or overweight women (104, 106-108). Alteration of the gut microbial environment by probiotics as a means to prevent metabolic outcomes associated with pregnancy, is likely to be a promising area (109).

***Control and treatment of gestational diabetes:*** Disagreement regarding the treatment of gestational diabetes is still due to the lack of a universal standard to define glucose intolerance during pregnancy (110). For this reason, individual studies have yielded different results and led to confusion about the efficacy and safety of gestational diabetes mellitus. Based on studies in this area, nutritional interventions along with precise monitoring of blood glucose levels are considered as a primary therapeutic option, and drug therapy, if diet alterations fail, will begin to control blood glucose levels. It is estimated that 70-80% of the cases could only be controlled by changing lifestyle (111).

Blood sugar should be measured four times a day: fasting blood sugar (after waking up) and one and two hours after eating each main meal (112). Recent recommendations for blood sugar targets patients with fasting blood sugar less than 96 mg/dl, sugar one hour after food lower than 140 mg/dl and less than 120 mg/dl two hours after a meal (23). There is no consensus on the timing of initiation of insulin therapy, but there are more conservative guidelines for reducing macrosomia and related risks in the fetus (112, 113). According to National Institute for Health and Care Excellence (NICE) in England, if the above mentioned goals are not met with the diet and lifestyle recommendations within 2 weeks, treatment should be started(114, 115). Standard drug for patients with gestational diabetes who require medication, insulin is available. However, since Langer et al. compared the use of insulin and glibenglamide in these patients, oral medications have been increasingly considered to be secondary treatments (116). Descriptive studies and clinical trials have examined the use of, mainly glibenglamide and metformin (117). Oral medications have been considered for ease of use and cost, and this has led to an increase in the use of glucose-lowering drugs, particularly metformin and glyburide have been implicated in pregnancy (118). According to Rowan and colleagues who compared the use of insulin and metformin in women with gestational diabetes, metformin is a safe option for the treatment of gestational diabetes and has a higher acceptability in patients (119).

***Nutritional interventions:*** Nutritional interventions in the diet are the most important treatment for gestational diabetes. In all recent workshops and conferences on diabetes mellitus (24, 120-123), MNT has been mentioned as the cornerstone of the treatment of gestational diabetes. Quantity and quality of Nutrition have an important role in the development of embryos. Specifically, the management of these patients involves manipulating and limiting calories and nutrients as a normalization strategy. MNT is identified as “designing meals with controlled carbohydrate levels for nutritional adequacy with normal nutrition, normal sugar levels and prevention of Ketosis” (124).

In a recent review on the effects of dietary interventions, lifestyle changes and dietary supplements on the prevention of gestational diabetes, it has been concluded that positive results have not been achieved in trials that have only been intercepted by participants in food intake. But dietary interventions with lifestyle interventions have been shown to be more effective in reducing the prevalence of gestational diabetes mellitus. Ameliorated outcomes consist of lesser birth weight and a decrease in incidence of macrosomia (125, 126), requirement for insulin medication (127), disorders of high blood pressure in pregnancy (126, 128), neonatal admissions to intensive care unit and deaths(125, 126). As stated above, the basis of work is based on proper nutrition (129).A skilled clinical nutritionist should provide MNT based on ordered and regular visits to women with GDM. 

 Calorie allocation is based on ideal body weight. The recommendations are 30 kilocalories per kilogram body weight in women with normal body mass index, 24 kilocalories per kilogram body weight in overweight women and 12-15 kilocalories per kilogram in women with obesity. While specialization of the calorie prescription, prepregnancy weight and body mass index (BMI), gestational weight gain, and physical activity should be considered (125). In terms of macronutrients, adequate amounts of macronutrients should be provided to support pregnancy, according to nutrition assessment, with directions from the DRIs.

The recommended daily energy intake of macronutrients is 33-40% complex carbohydrates, 35-40% fat and 20% proteins (113). It has been shown that the calorie intake, insulin function and metabolism status in obese diabetic patients are improved (130). There is limited data on the correlation between calorie intake and gestational control in pregnant women, and there is little evidence for this in terms of quality (131). In general, it is assumed that due to lack of proper control of blood glucose in pregnant mothers, the low dependence to dietary recommendations and calorie intake is more than real need (132). The general approach in these patients is to restrict or modify calorie intake before starting insulin therapy. However, this self-restraint in the diet can have unwanted effects on the diet and weight gain of the pregnant mother (133). Receiving diet without professional advice, despite the proper design of pregnant women with gestational diabetes or type 2 diabetes, has never been desirable due to the possibility of ketoacidosis during pregnancy, which has a great risk to mother and fetus. Mean calorie restriction (33% reduction in calorie intake) does not lead to ketosis, but controls weight gain and glucose levels in obese women (134).

Carbohydrates are the most important nutrient that affects glucose levels after meals. Carbohydrate intake can be manipulated through total intake of carbohydrates in a daily diet, carbohydrate distribution in the main meals, snacks and carbohydrates (135). As gestational diabetes is a type of glucose intolerance, learning about carbohydrate foods is vital to facilitate food choices. The conventional approach to restrict dietary carbohydrates (in the case of preserving dietary protein in the range of 15-20% of daily calorie intake) leads to an increase in fat intake in a daily diet (136). DRI report has considered at least 175 g glucose per day CHO, a minimum of 71 g protein (or 1.1 g/kg/day), and 28 g fiber for pregnant women(99). Another important factor in addition to its carbohydrate content, is a glycemic index (GI) that categorizes carbohydrates based on the ability to increase blood sugar according to glucose or white bread (137), because ih has been observed that different nutrients with similar carbohydrate content have different effects on blood glucose levels in patients (96, 138-141). Now, this index as a potential tool for designing a diet for diabetic patients has a key role in preventing and managing diabetes (142, 143).

Based on the results of a systematic review and meta-analysis to determine the effect of a low-glycemic diet on gestational diabetes, a low-glycemic diet diminishes the risk of macrosomia in affected patients, which is apparent in association with a high-fiber diet. Based on the results, the recent diet (low-glycemic plus high-fiber diet) greatly reduces the need for insulin in diabetic patients (144).

***Exercise and Gestational Diabetes:*** Exercise helps overcome insulin resistance and control fasting and post-meal hyperglycemia, and may be used as a supplement to nutritional interventions to improve the blood glucose levels in mother. The most ideal type of exercise is not known, but it is often recommended to walk fast after the main meals (145).

***Diet supplements:*** Today, public health and knowledge has grown and people are looking for ways to prevent disease and promote their health. Diet supplements include various macronutrient and micronutrient sources in different forms of capsules, pills, syrups, powders, granules, soft gels, oral drops and …, which have a fixed and consistent composition for use in human and, in some circumstances, compensating nutrient supplements are useful and necessary (146).

***Vitamin D: ***Recently, much attention has been paid to the role of vitamin D in controlling insulin sensitivity. Various animal studies (147, 148) and human (149, 150) support the role of vitamin D in secretion and insulin dysfunction and increase insulin tolerance through multiple mechanisms. The effect of vitamin D supplementation in healthy subjects (151, 152) and type 2 diabetes patients (153) has shown a decrease in insulin resistance. Recent trials have shown that in the first and second trimesters of pregnancy vitamin D supplementation reduces the risk of intolerance to glucose and gestational diabetes in the third trimester (154).

According to the results of Zhang et. al, supplement treatment with high dose of vitamin D (50 thousand units every two-week) improves insulin resistance in women with gestational diabetes mellitus (155).

***Inositols: ***Inositol belongs to the group of vitamins in group B-complex and the main source is diet. The 6-hydroxyl-inositol-6-hydroxyl-group hydrolysis of the DCI and MYO groups is included in the stero-isomer formulation 9, both of which are used as insulin-susceptible drugs (156). Inositol is naturally present in cereals, maize, legumes and meat, and is essentially made in the liver (157).

***Myo-Inositol: ***Several clinical trials have been conducted on the effects of myo-Inositol supplement on the prevention of gestational diabetes. In the study of D'Anna and colleagues, myo-Inositol supplementation in pregnant women with a family history of type 2 diabetes, with no side effects, reduced the incidence of gestational diabetes and decreased birth weight in adolescents receiving myo-Inositol in comparison with placebo (147). The administration of myo-Inositol supplement for 8 weeks in pregnant women with a new diagnosis of gestational diabetes has led to a decrease in serum levels of insulin and glucose (158).

***Fish oil (Omega-3): ***The useful impacts of omega-3 intake on glucose and lipid metabolism and inflammatory indices in GDM have been shown in recent studies (159, 160). Anderson et al.(161)suggested that supplementation with 3.4 g/day eicosapentaenoic acid (EPA) and docosahexaenoic acid (DHA) for 2–3 weeks upregulats gene expression of PPAR-γ. Further, omega-3 consumption has been revealed to enhance gene expression of adiponectin bymodifying the transcription factor PPAR-γ(162, 163). A substantialenhancement in gene expression of PPAR-γ in pup mice’s brain was also perceivedsubsequent the prescription of omega-3 (164).Moreover, Omega-3 consumption might reduce inflammatory cytokine releases by preventing the activation of NF-кB (165). 

***Probiotics: ***Due to the importance of the intestinal microbial population in the development of diseases associated with dysbiosis, interest in the treatment of microbial environment in the intestine, including the use of probiotics, has recently increased (166, 167). Objectives of special probiotic interventions in gestational diabetes include the correction and normalization of native microbial characteristics, intestinal dysfunction and immune regulation for better control of local and systemic inflammation (168). Probiotic species that have been most promising include members of the Lactobacillus family, Bifidobacterium and Enterococcus (98). The minimum effective dose of probiotics for a single-physiological effect is observed in the daily doses of 108-1010 unit colony producing. Regarding the mechanism of glucose-lowering effects of probiotics, controversial reports of immuno-regulatory and anti-inflammatory properties of probiotics have been presented in the studies (169, 170). Probiotics are probably the missing element of dietary interventions that focus on how the food matrix and dietary contents interact with the intestinal microbiota. Therefore, specific probiotics, along with dietary interventions, may control intestinal dysfunction, local and systemic inflammation and inappropriate metabolic regulation during pregnancy (168, 171-173).

***Prebiotics: ***The term “prebiotic” refers to the components of the diet (mainly non-digestible oligosaccharides), which selectively stimulate the growth and activity of a limited number of microbial species and strains (174). Studies have shown that intestinal microbiota can be modulated by administering inulin fructans and galactans with a predominant effect on bifidobacteria and to some extent on lactobacillus species (175).

In some human and animal studies, it has been shown that prebiotics decrease the amount of intestinal microbial enzymes, energy intake and body weight (176, 177) and simultaneously reduce insulin resistance and hyperglycemia (178-180). These effects appear to be due to increased secretion of intestinal hormones of Pyy, GLP-2 and GLP-1 (158,162), reduced appetite peptide of avergerlin (177, 181) and endotoxemia reduction by improving the function of the mucous membrane and reducing the level of inflammatory markers (180, 181). The direct effect on the production of short fatty acids (butyrate) is another potential mechanism that the use of prebiotic in this way can have a beneficial effect on host physiology (182, 183).

## Conclusion

Finally, it can be concluded that gestational diabetes mellitus is a growing health problem in the world and is one of the most common complications of pregnancy (24) and one of the causes of the epidemic type 2 diabetes in the world (184, 185). Pregnancy is likely to be a critical period for appropriate interventions and actions aimed at reducing the incidence of type 2 diabetes (6).

The Western lifestyle, along with the increasing prevalence of obesity globally, has led to an increase in the weight of pregnant mothers. Overweight and obese pregnant women are at increased risk for pregnancy complications such as gestational diabetes mellitus. Treatment of patients with gestational diabetes mellitus provides an ideal context for primary interventions to prevent type 2 diabetes. Treatment and nutritional intervention are the primary treatment for gestational diabetes mellitus and obesity is a major challenge in patient counseling and interventions during pregnancy (186). In fact, the effects of maternal nutrition during pregnancy on a child may initiate a cascade of metabolic and inflammatory immune events that appear in later stages of life. Therefore, nutritional environments in this period of time provide an opportunity to reverse the growing trend of diseases associated with Western lifestyle and, as such, attract the increasing attention of nutrition scientists.
